# Scavenger Receptor Class B Type 1 (SR-B1) being a Potential Biomarker for the Diagnosis of Liposarcoma and Associated with the Degree of Differentiation of Liposarcomas

**DOI:** 10.7150/jca.31730

**Published:** 2019-07-10

**Authors:** Qianqian Liu, Zhenzhen Li, Hao Shang, Qiaochu Zhang, Xiaomeng Wang, Yangyang Zhang, Yang Wang, Qianru Li, Chunsen Li, Chunxia Liu, Feng Li

**Affiliations:** 1 Shihezi University, Shihezi 832002, Xinjiang, P.R. China. a. Department of Pathology, School of Medicine. b. The Key Laboratories for Xinjiang Endemic and Ethnic Diseases, Chinese Ministry of Education. c. The First Affiliated Hospital, School of Medicine.; 2 Department of Pathology, Beijing Chaoyang Hospital, Capital Medical University, Beijing 100020, P. R. China.

**Keywords:** Scavenger receptor class B type 1, Immunohistochemistry, Tissue microarray, Liposarcoma, Biomarker

## Abstract

**Background**: Soft tissue sarcomas include multiple histological subtypes and are highly aggressive. Moreover, SR-B1 is associated with malignant behavior and poor prognosis in a variety of cancers. However, there have been no attempts to assess whether SR-B1 expression in soft tissue sarcoma. We aimed to detect the expression levels of the SR-B1 protein in soft tissue sarcoma.

**Methods**: We assessed SR-B1 expression via immunohistochemistry and tissue microarrays in 107 soft tissue sarcomas with 4 phenotypes: 26 liposarcomas, 18 Ewing's sarcomas, 20 rhabdomyosarcomas and 43 leiomyosarcomas.

**Results**: Tumor cell SR-B1 expression was seen in 18/26 (69.2%) liposarcomas, 1/18 (5.55%) Ewing's sarcomas, 1/20 (5.00%) rhabdomyosarcomas, 2/43 (4.70%) leiomyosarcomas and was stained in the cell membrane. We found that SR-B1 expression in liposarcomas (18/26) was significantly higher than that in non-lipomatous sarcomas (4/77) (*χ*2 = 49.811, *p* = 0.000) and in well-differentiated liposarcoma (13/15) was significantly higher than that in dedifferentiated liposarcoma (5/11) (*p* = 0.038). No significant correlation was found between SR-B1 and gender, nationality, size and tumor location (*p* > 0.05), but it was significantly associated with ages (*χ*2 = 11.426, *p* = 0.001) and sarcoma phenotypes (*χ*2 = 49.817, *p* = 0.000).

**Conclusion**: Our findings highlight the highly expression of SR-B1 in liposarcomas. SR-B1 may be a potential biomarker for the diagnosis of liposarcoma and may indicate the degree of differentiation of liposarcomas.

## Introduction

Soft tissue sarcomas are a rare and highly complex group of neoplasms, and can originate from almost anywhere in the body 1, 2. Significantly, soft tissue sarcomas contain multiple histological phenotypes, and are highly invasive 3, posing significant challenges in diagnostic and clinical treatment. The clinical and imaging findings of soft tissue sarcomas are similar; therefore, diagnosis with histopathology is necessary. Even with multimodality treatments that involve surgery, radiotherapy, and combination chemotherapy, more than 40% of patients have suffered from tumor recurrence, which leads to an overall survival of less than 12 months with stage III metastases 4. Therefore, because the diagnosis and treatment of soft tissue sarcoma is a major challenge for clinicians, novel targeting targets are needed for treating and identification soft tissue sarcomas.

Previous studies have shown that CD36 is highly expressed in liposarcoma and low expressed in rhabdomyosarcoma, and have proposed that CD36, a cell surface molecule, may be related to the phenotype of soft tissue tumors 5, 6. Zhang Y et al. found that CD36 expression in well-differentiated liposarcomas was significantly higher than in differentiated liposarcoma 6, and have proposed that CD36 may be related to liposarcomas differentiation. In 1993, Calvo and Vega first identified a kind of cell-surface glycoprotein, named scavenger receptor class B type 1 (SR-B1), which belongs to the CD36 superfamily of membrane-bound cells 7. Previous reports have indicated that SR-B1 is highly expressed in numerous tumor cells, such as in hepatoma, prostate, breast, colorectal, pancreatic, ovarian, and nasopharyngeal cancers 8-11. Furthermore, the overexpression of SR-B1 accelerates the uptake of high-density lipoprotein-cholesterol ester and the binding of high-density lipoproteins, which are important nutrients in the proliferation and metastasis of malignant cells 12-14. Some studies have pointed out that high-density lipoprotein level is negatively correlated with the risk of cancer development and metastasis 15-17. Su et al. has revealed that high-density lipoprotein mimetic peptides can inhibit tumor growth 18, 19. Moreover, Ng et al. and Zhang et al. have suggested that high-density lipoprotein-mimicking peptide-phospholipid scaffold (HPPS) nanocarriers exhibit high binding affinity to SR-B1-expressing cancer cells 20, 21. Furthermore, Zheng et al. have identified SR-B1 as a potential biomarker, and that the use of HPPS as an effective anti-nasopharyngeal carcinoma agent may shed new light on the diagnosis and therapeutics of nasopharyngeal carcinoma 11.

As a protein in the CD36 superfamily, the use of SR-B1 as a potential biomarker for diagnosing and treating various human epithelial tumors has been extensively studied. However, studies on the diagnosis and treatment of SR-B1 in human soft tissue sarcoma have not been systematically clarified. In this study, we used tissue microarray and immunohistochemistry to detect the expression levels of SR-B1 in 107 cases of 4 different phenotypes of soft tissue sarcoma to evaluate the clinical relevance of different sarcoma phenotypes. Our study was designed to detect the expression levels of the SR-B1 protein in liposarcomas and non-lipomatous sarcomas to provide new insights into the diagnosis, identification, and clinical treatment of soft tissue sarcomas.

## Material and Methods

### Patients and tissue specimens

A total of 107 formalin-fixed and paraffin-embedded soft tissue sarcoma specimens were obtained from the Department of Pathology of The First Affiliated Hospital of Shihezi University School of Medicine, China. All specimens were anonymized, approved, and supervised in accordance with the policies of the Research Ethics Committee of The First Affiliated Hospital of Shihezi University School of Medicine. Clinical, demographic, and pathologic data were acquired from the medical records of the patients. A histopathologic evaluation was conducted independently by 2 pathologists. All histological types of tumor and histologic grading were retrospectively reviewed in the 107 cases according to the 2013 World Health Organization classification of tumors of soft tissue and bone 3. We studied archival materials of 26 liposarcomas, 18 Ewing's sarcomas, 20 rhabdomyosarcomas, 43 leiomyosarcomas and 19 normal adipose samples. Among the 26 liposarcomas, 15 well-differentiated liposarcomas and 11 dedifferentiated liposarcomas were included in this study.

### Tissue microarrays construction

Tissue microarrays were constructed in accordance with established methods 22. The same patient has only primary tumor tissue for the microarray, and multiple samples of the same patient are not repeated. One representative paraffin-embedded tissue block was selected from each patient, and one cores were taken from morphologically representative areas of the block. After reviewing original hematoxylin and eosin slides, tissue microarrays were established from the representative fields of tumors from the paraffin-embedded tissue blocks. A typical area (3 mm in diameter) was taken from each paraffin block (donor block) and arranged in a tissue array using a ring drill. All tissue microarrays blocks were confirmed via H&E staining. Finally, sections were continuously sliced from tissue microarrays (4 μm thick) for immunohistochemical staining.

### Immunohistochemical staining and assessment

Antigen retrieval was performed using Tris-EDTA buffer (pH 9.0). Sections were immunostained using monoclonal rabbit antihuman antibody against SR-B1 (clone: ab52629, 1:250 dilution; Abcam, Burlingame, CA). The EnVision System (Dako, Carpinteria, CA) and 3, 3′-diaminobenzidine peroxidase substrate kit (Dako, Carpinteria, CA) were used to detect antibody-conjugated peroxidase activity. The sections were then counterstained with hematoxylin, dehydrated, and mounted. PBS was used instead of the primary antibody as the negative control. Membranous immunoreactivity for SR-B1 protein was scored semi-quantitatively by calculating the percentage of positive cells and staining intensity. The immunostained tissues were blindly reviewed and scored under a light microscope by two pathologists that were blinded to the clinicopathological characteristics and survival data. In cases of discrepancy, a consensus was made through an in-depth discussion on multi-head microscopic observations. No consensus was reached regarding the scoring system for SR-B1 expression, especially in soft tissue sarcoma; thus, we modified the previous protocol and described the SR-B1 expression with as much detail as possible by using a semi-quantitative manner using the intensity multiplied by the proportion. The average staining intensities of tumor cells were scored as: 0, no staining; 1, weak; 2, moderate; and 3, strong. The staining percentage was scored as: 0, < 5%; 1, 6%-25%; 2, 26%-50%; 3, 51%-75%; 4, > 75%, respectively. Specimens were evaluated as positive if the sum score was ≥ 2, with 1+ (2-4 points), 2+ (5-8 points), 3+ (9-12 points).

### Statistical methods

All statistical analyses were performed using SPSS 20.0 (IBM, Chicago, IL, USA). The correlations between SR-B1 expression and clinicopathologic variables were analyzed using the independent sample t-test for the continuous variables and the chi-square test for the discrete variables. In all statistical analyses, a *p*-value of less than 0.05 was considered statistically significant.

## Results

**Figure 1:**
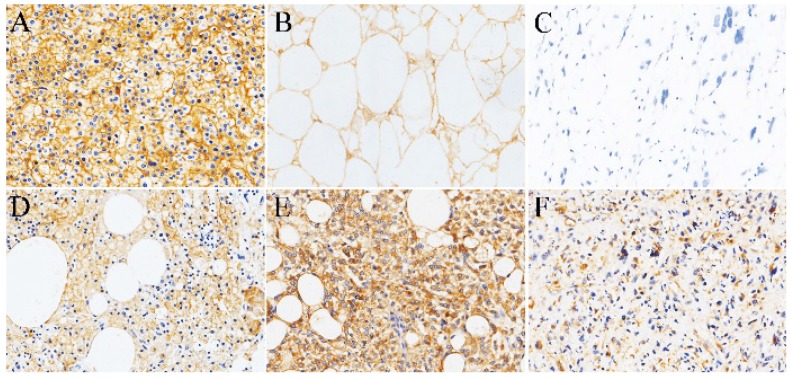
** Representative immunohistochemical staining of SR-B1 in normal adipose tissues and several subtypes of liposarcomas.** (A) Positive control of SR-B1 in normal liver tissue. (B) Normal adipose tissues with membrane-positive immunostaining. SR-B1 staining is primarily observed in liposarcoma cell membranes. (C) Negative SR-B1 staining is shown in liposarcoma tissue (scored as negative). (D) and (E) show moderate and strong SR-B1 staining in well-differentiated liposarcoma tissues, respectively (scored as 1+ and 2+, respectively). (F) represent the positive expression of SR-B1 in dedifferentiated liposarcomas. (×200 magnification). SR‑BI, Scavenger receptor class B type I.

Tumor specimens from 107 patients with soft tissue sarcoma were subjected to immunohistochemical analysis, 53 (20.5%) of them showed positive expression levels of SR-B1, with 19 cases showing 1+ positivity and 3 cases showing 2+ positivity. The immunohistochemical staining results of the SR-B1 protein levels are summarized in Table 1. The detailed study of SR-B1 in each sarcoma is as follows:

**Normal adipose tissue and Liposarcoma.** As a positive control, Fig. 1A shows diffuse strong positive expression of SR-B1 in the cell membrane of the normal liver tissue cells. Our study found that adult non-tumor human normal fat showed high SR-B1 protein expression on the cell membranes of normal adipocytes. A total of 14 (73.7%) of the 19 adult non-neoplastic normal adipose samples tested showed positive expression levels of the SR-B1 protein, and a clear image is shown in Fig. 1B. We observed that the expression of SR-B1 protein in most non-tumor normal adipose tissue was mainly concentrated in 1+ and 2+, and only one expression level was 3+. Of the 26 liposarcomas samples analyzed, 18 showed SR-B1 protein expression in tumor cells (69.2%), which of 13/15 (86.7%) in well-differentiated liposarcoma, 5/11 (45.5%) in dedifferentiated liposarcomas cases showing SR-B1 protein expression, and full details are shown in Table 2. More importantly, the expression of SR-B1 protein in well-differentiated liposarcoma was significantly higher than that in dedifferentiated liposarcoma (*p* = 0.038). SR-B1 protein was expressed in the cell membrane of liposarcoma cells. Positive and negative expression levels of SR-B1 protein in liposarcoma are shown in Fig. 1C-1F. A total of 14 patients with liposarcoma showed an immunohistochemical score of 1+, and 4 patients showed a score of 2+, as shown in Fig. 1D and Fig. 1E, respectively. We analyzed the associations between SR-B1 protein expression and several clinicopathological parameters of liposarcoma patients (Table 3). No significant correlations were observed between SR-B1 expression and clinicopathological parameters, including patient age (≤48 years versus >48 years), gender, ethnicity, tumor diameter (≤5cm versus >5cm), tumor location (*p* >0.05).

**Figure 2:**
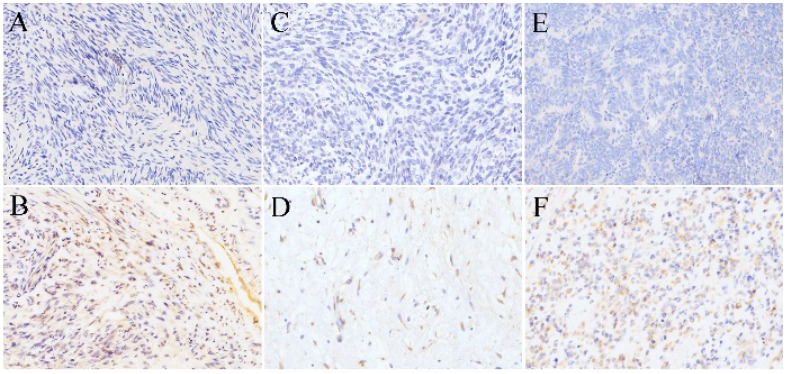
** SR-B1 expression in non-lipomatous sarcomas.** (A), (C) and (E) show negative expression of SR-B1 in leiomyosarcoma, rhabdomyosarcoma and Ewing's sarcoma, respectively. There was no positive staining of SR-B1 in the cell membrane, nucleus and cytoplasm of tumor cells. (B), (D) and (F) show weak SR-B1 staining in leiomyosarcoma, rhabdomyosarcoma and Ewing's sarcoma, respectively. (×200 magnification).

**Leiomyosarcoma.** Leiomyosarcoma had the lowest expression of SR-B1 protein, and only 2 of 43 leiomyosarcomas were showed an immunohistochemical score of 1+ (4.70%), no expression of SR-B1 was found in 41/43 (95.34%) cases of leiomyosarcoma. In Fig. 2A, there was no SR-B1 staining in the cell membrane, nucleus and cytoplasm of most leiomyosarcomas. Fig. 2B shows that SR-B1 protein is expressed in the leiomyosarcoma cell membrane and shows weak positive staining.

**Rhabdomyosarcoma.** There were 20 rhabdomyosarcomas cases, 9 cases of embryonal rhabdomyosarcomas and 9 cases of alveolar rhabdomyosarcomas, and 2 cases of pleomorphic rhabdomyosarcomas. The positive expression of SR-B1 protein in rhabdomyosarcoma was 1/20 (5.00%), and negative expression was 19/20 (95.0%) (Fig. 2C). In 20 rhabdomyosarcoma cases, no diffuse strong positive expression of SR-B1 protein was found, and only 1 cases alveolar rhabdomyosarcoma showed localized weak staining. The SR-B1 protein expression shown in Fig. 2D is contained in tumor cells, in which the SR-B1 protein is weak stained in the cell membrane of rhabdomyosarcoma cells.

**Ewing's sarcoma.** Of the 18 Ewing's sarcoma samples analysed, 1 (5.55%) had SR-B1 expression in tumor cells, staining was weak in 1+, 17 (94.4%) had not SR-B1 expression in tumor cells (Fig. 2E). As shown in Fig. 2F, the cell membrane of less than 40% of the tumor cells showed SR-B1 expression in one weak leiomyosarcoma.

### Clinicopathological features

To assess SR-B1 expression in soft tissue sarcomas, we analyzed the relationship between SR-B1 protein and the clinicopathological parameters of the patients with soft tissue sarcoma, respectively (Table 4). We found that patients with soft tissue sarcoma with ages (*χ*2 = 11.426, *p* = 0.001) and sarcoma phenotypes (*χ*2 = 49.817, *p* = 0.000) exhibited reduced SR-B1 expression, whereas SR-B1 expression was not associated with gender (*χ*2 = 1.050, *p* = 0.305), ethnicity (*χ*2 = 0.000, *p* = 1.000), tumor diameter (*χ*2 = 0.366, *p* = 0.545) and tumor locations (*χ*2 = 6.704, *p* = 0.082). More importantly, we found that the expression of SR-B1 in liposarcomas was significantly higher than that in non-lipomatous sarcomas (*χ*2 = 49.811, *p* = 0.000), and full details are shown in Table 5.

## Discussion

SR-B1 a kind of cell-surface glycoprotein that belongs to the CD36 superfamily of membrane-bound cells. SR-B1 is well conserved in species and is expressed in many mammalian tissues and cell types, including the intestines, macrophages, endothelial cells, smooth muscle cells, keratinocytes, adipocytes, and the placenta. Significantly, SR-B1 is highly expressed in tissues that are dependent on high-density lipoprotein free cholesterol and cholesteryl ester for bile acid synthesis (liver) and steroidogenesis (adrenal, ovary, and testis) 23. Furthermore, SR-B1 promotes tumor cell proliferation and metastasis by regulating lipid metabolism 3, 4, 13.

SR-B1 is highly expressed in various epithelial-derived tumor cells, and immunohistochemical assessment of scavenger receptor proteins is helpful as a prognostic marker for various cancers. Moreover, blocking scavenger receptor proteins may help prevent cancer cell metastasis 12, 15, 16, 24.

Previous reports have addressed the expression of CD36 in liposarcomas 6, and our work provides information regarding the expression of SR-B1 in various phenotypes of soft tissue sarcomas, in order to distinguish between liposarcomas from non-lipomatous sarcomas. Consistent with previous studies, we found that SR-B1 protein is stained in the cell membrane of normal adipocytes and soft tissue sarcoma cells. Mechtersheimer G et al. detected the positive expression rates of CD36 in 4/11 (36.4%) liposarcoma, 2/8 (25%) rhabdomyosarcoma 5. Our study has demonstrated that SR-B1 protein not only was high expressed in 18/26 (69.2%) liposarcoma, but also was low expressed in 1/20 (5.00%) rhabdomyosarcoma, 2/43 (4.70%) leiomyosarcoma and 1/18 (5.55%) Ewing's sarcoma. In addition, our study demonstrated for the first time that the expression of SR-B1 was significantly higher in liposarcomas than in non-lipomatous sarcomas (*p* = 0.000), suggesting that SR-B1 may serve as a biomarker for distinguishing between liposarcomas from non-lipomatous sarcomas. The high expression of SR-B1 was significantly associated with age (*p* = 0.001) and sarcoma phenotypes (*p* = 0.000). Additionally, Zhang Y et al. found that CD36 is a marker of adipocyte differentiation in liposarcoma tissues and cells, and its expression in well-differentiated liposarcomas is significantly higher than in dedifferentiated liposarcomas 6. Consistent with the results of Zhang et al, we also found that the expression of SR-B1 in well-differentiated liposarcomas was significantly higher than in dedifferentiated liposarcoma (*p* = 0.038), which suggest that SR-B1 may be a marker for differentiation of liposarcomas.

SR-B1 is involved in cholesterol transportation. It is a high density lipoprotein receptor that mediates selective uptake of the lipid from high density lipoprotein, which initially indicated that SR-B1 can play a critical role in high density lipoprotein metabolism 25. Studies have showed that during SR-B1-dependent selective cholestery l ester uptake, caveolin-1 is a negative regulator 26. Moreover, recent studies have shown that SR-B1 is localized in the caveolae numerous cell types, and SR-B1 colocalizes with caveolin-1 27, 28. Additionally, caveolae are especially abundant in adipose tissue, playing a consistent role in a number of processes, such as the insulin-dependent glucose uptake, transmembrane transport of lipids underlying differentiation, and maintenance and adaptive hypertrophy of adipocytes 29. The analysis likely indicates that caveolin-1, caveolin-2, and the caveolin-1 cluster in each liposarcoma histotype, reaching maximal expression in well-differentiated tumors 24, it was consistent with our study, which may explain that the expression of SR-B1 in liposarcoma is higher more than other three non-lipomatous sarcomas, especially in well-differentiated liposarcoma. Further studies have found that the expression of caveolin-1 is not only related to the degree of tumor cell differentiation, but also may represent a key step in the progression of liposarcoma tumorigenesis 24. In conclusion, SR-B1 and caveolin-1 may be co-located in liposarcoma, especially well-differentiated liposarcoma, which is related to the degree of cell differentiation of liposarcoma. More importantly, SR-B1 and caveolin-1 protein may be play a role in the occurrence and development of liposarcoma together. SR-BI has been implicated as a mediator of several cell-signaling events in the context of atherosclerosis. Distinct ligands binding to SR-B1 results in some intracellular signal events that mainly include varying the activity of signal molecules in the PI3K/Akt and MAPK pathways 30-32. Activation of the PI3K/Akt and MAPK pathway promotes tumor growth, survival, and proliferation and has been implicated in a variety of human cancers, including liposarcoma, rhabdomyosarcoma, Ewing's sarcoma, and fibrosarcomas 33, 34.

In conclusion, we have identified SR-B1 as a potential biomarker with diagnosis values in liposarcoma, which was significantly increased in liposarcoma, compared with rhabdomyosarcoma, leiomyosarcoma and Ewing's sarcoma. Moreover, we hypothesize that SR-B1 may indicate the degree of differentiation of liposarcomas, which in well-differentiated liposarcoma was significantly higher than that in dedifferentiated liposarcoma. Further studies in biological function are needed to validate our findings.

## Figures and Tables

**Table 1:** Immunohistochemical expression of SR-B1 in normal adipose tissue and soft tissue sarcoma tissue microarrays.

Tissue types	SR-B1 expression			Total positive
	1+	2+	3+	
Normal adipose tissue	10	3	1	14/19 (73.7%)
Liposarcoma	15	3	0	18/26 (69.2%)
Rhabdomyosarcoma	1	0	0	1/20 (5.00%)
Leiomyosarcoma	2	0	0	2/43 (4.70%)
Ewing's sarcoma	1	0	0	1/18 (5.55%)

**Table 2:** Semi-quantitative assessment of SR-B1 protein in different subtypes of liposarcomas.

Liposarcoma subtypes	SR-B1 expression			Total positive	*p*-value
	1+	2+	3+		
Well-differentiated liposarcoma	10	3	0	13/15 (86.7%)	0.038*
Dedifferentiated liposarcoma	4	1	0	5/11 (45.5%)	

**Table 3:** Relationship between SR-B1 expression and clinicopathological parameters in liposarcoma.

Clinicopathological Variables	Total case	SR-B1 expression		*p*-value
		negative	positive	
Age				1.000
≤48 years	5	1	4	
>48 years	21	6	15	
Gender				1.000
Male	15	5	10	
female	11	3	8	
Ethnicity				1.000
Han	20	6	14	
Other minorities	6	2	4	
Tumor diameter				1.000
≤ 5cm	6	2	4	
>5cm	20	6	14	
Tumor location				0.322
Head/Neck	2	0	2	
Extremities/Trunk	11	4	7	
Thorax/Abdomen/Pelvis	12	3	9	
Genitourinary	1	1	0	

**Table 4:** Relationship between SR-B1 expression and clinicopathological parameters in soft tissue sarcoma.

Clinicopathological Variables	Total case	SR-B1 expression		χ2 (*p*-value)
		negative	positive	
Age				11.426 (0.001)*
≤48 years	63	57	6	
>48 years	44	28	16	
Gender				1.050 (0.305)
Male	48	36	12	
Female	59	49	10	
Ethnicity				0.000 (1.000)
Han	168	121	47	
Other minorities	29	23	6	
Tumor diameter				0.366 (0.545)
≤ 5cm	40	33	7	
>5cm	67	52	15	
Tumor location				6.704 (0.082)
Head/Neck	9	6	3	
Extremities/Trunk	29	22	7	
Thorax/Abdomen/Pelvis	40	30	10	
Genitourinary	28	27	1	
Sarcoma phenotypes				49.817 (0.000)*
Liposarcoma	26	8	18	
Rhabdomyosarcoma	20	19	1	
Leiomyosarcoma	43	41	2	
Ewing's sarcoma	18	17	1	

**Table 5:** Semi-quantitative assessment of SR-B1 protein in liposarcomas and non-lipomatous sarcomas.

Sarcoma phenotypes	SR-B1 expression			Total positive	χ2 (*p*-value)
	1+	2+	3+		
Liposarcoma	15	3	0	18/26 (69.2%)	49.811 (0.000)*
Non-lipomatous sarcoma	4	0	0	4/81 (4.9%)	

## Abbreviations

SR-B1: Scavenger receptor class B type 1
CD36: cluster of differentiation 36
PPARα/γ: peroxisome proliferator activated receptor
RCT: reverse cholesterol transportation
LH: luteinizing homone
HCG: human chorionic gonadotrop(h)
INFα: interferonα
IGF-1: insulin-like growth factor
PI3K: Phosphatidylinositol (-3) kinase
MAPK: Mitogen-activated protein kinase
eNOS: nitric oxide synthase
